# Assessment of right atrium dysfunction in patients with obstructive sleep apnea syndrome using velocity vector imaging

**DOI:** 10.1186/s12947-018-0150-y

**Published:** 2018-12-13

**Authors:** Junfang Li, Changhong Lu, Wugang Wang, Kun Gong, Liang Zhao, Zhibin Wang

**Affiliations:** 1 Department of Echocardiography, Qingdao University Affiliated Hospital, No. 16 Jiangsu Road, Qingdao, 266001 China; 2 Department of Heart Center, Qingdao Fuwai Cardiovascular Hospital, Qingdao, 2660034 China

**Keywords:** Velocity vector imaging, Obstructive sleep apnea syndrome, Right atrial strain, Right atrial function

## Abstract

**Background and Objectives:**

This study aimed to assess the changes of RA function in patients with obstructive sleep apnea syndrome (OSAS) using velocity vector imaging (VVI) and to evaluate the application of VVI technology.

**Methods:**

According to the apnea–hypopnea index (AHI), 71 patients with OSAS were divided into three groups: mild, moderate, and severe. A total of 30 cases of healthy subjects were enrolled as the control group. Digital images of apex four-chamber views were acquired to measure the right atrium (RA) linear dimensions and volume parameters including RA longitudinal diameter (RAL), transverse diameter (RAT), RA maximum volume (Vmax), RA minimum volume (Vmin), right atrial volume before contraction (Vpre). Right atrial volume parameters were corrected by body surface area (VImax, VImin, VIpre). The total right atrial emptying fraction (RATEF), right atrial passive emptying fraction (RAPEF), right atrial active contraction emptying fraction (RAAEF) were calculated. The VVI data measuring right atrial global strain (RA-GLS), right atrial strain rate in ventricular systolic phase (RA-SRs), right atrial strain rate in ventricular early diastolic phase (RA-SRe), right atrial strain rate in ventricular late diastolic phase (RA-SRa).

**Results:**

1. RA linear dimensions and volume parameters in severe OSAS were higher than those of control group. RAPEF in severe group was lower than control group and mild OSAS group (*t =* 2.681, *P* = 0.021; *t =* 2.985, *P* = 0.011; respectively). RAAEF in OSAS moderate group was higher than that of control group (*t =* 3.006, *P* = 0.02), and without statistical difference (*P* > 0.05) in the severe OSAS group and the control group.
2. RA-GLS in moderate OSAS group was significantly lower than that of control group (*t =* 2.333, *P* = 0.040) and reduced more obvious in the severe OSAS group (vs control, *t =* 3.25, *P* = 0.008, vs mild; *t =* 3.011, *P* = 0.012; respectively). RA-SRe in moderate and severe OSAS groups were lower than control group (*t =* 2.466, *P* = 0.031; *t =* 3.547, *P* = 0.005; respectively). RA-SRs of OSAS in severe group was lower than that of control and mild groups (*t =* 3.665, *P* = 0.004; *t =* 3.204, *P* = 0.008; respectively). RA-SRa in severe OSAS group was lower than that of control group (*t =* 2.425, *P* = 0.034).
3. Multivariate regression analysis showed that RA-GLS and RA-SRe were independently correlated with AHI (*t =* − 2.738, *P* = 0.010; *t =* − 2.191, *P* = 0.036; respectively).

**Conclusion:**

RA function was impaired in patients with OSAS. On hemodynamics, the change of RA function performed increased of reserve function, reduced pipeline function and increased of contraction function. However, the strain and strain rate reduced in different degree. RA-GLS and RA-SRe decreased the earliest, which suggested that strain and strain rate were the parameters which can reflect myocardial function damage earliest. VVI can more earlier and accurately detect myocardial dysfunction of right atrium in patients with OSAS, which is expected to be a worthy technique for early clinical therapy in patients with OSAS.

## Introduction

Obstructive sleep apnea syndrome (OSAS) is a common clinical disorder characterized by recurrent apnea and hypopnea during sleep caused by partial or complete upper airway collapse [1]. Patients with OSAS always complain of snoring, sleep fragmentation, waking up with a choking sensation, excessive sleepiness, unrefreshing sleep, fatigue or tiredness, and morning headache. The prevalence of OSAS is as high as 4% [2]. Because of its high prevalence and negative effect on the quality of life, OSAS is considered as a global health problem.

OSAS may lead to hypertension, pulmonary hypertension (PH) and associates with a series of cardiovascular disorders, including coronary artery disease and right ventricular (RV) dysfunction [3, 4]. The RV dysfunction may increase RV end-diastolic pressure, right atrial pressure (RAP), leading to RA dilatation and eventually right heart failure. Studies confirmed that the RA enlargement is an independent predictor of mortality and prognosis in patients with PH [5]. Recent studies have shown that the sensitivity and specificity of the RA morphology is higher than that of RV in predicting clinical events [6]. However, RA function in patients with OSAS has not yet to be fully elucidated.

The basic functions of the RA include: 1. Reservoir function, the function of storing blood at the time of RV contraction. 2. Conduit function, acting as a conduit of blood from the vein to the RV in the early ventricular diastolic time. 3. Contraction function, contracting actively to increase RV filling in the late ventricular diastolic time [7]. The volume produced by the RA contraction account for about 15–25% of the whole RV filling volume, and increase along with age and heart rate.

Magnetic resonance imaging (MRI) can be used to assess the RA function [8], but it is impossible to assess the RA regional function using MRI. Two-dimensional echocardiography (2DE), pulsed Doppler and tissue Doppler echocardiography also can be used to assess the RA function. However, these parameters are less reliable because of the influence of loading conditions and the angle of examination. Therefore, accurate assessment of RA function remains a challenge. Two-dimensional strain has been applied to the evaluation of cardiac function [9]. The RA function has been evaluated by 2-dimensional speckle-tracking echocardiography (2D-STE) in patients with PH [10], RV myocardial infarction [11] and healthy subjects [12]. But there is no reports about studies of the RA function in patients with OSAS. The velocity vector imaging (VVI) method is a unique technology that assesses myocardial strain (S), and strain rate (SR) based on grayscale (B-mode) images. VVI is independent of beam angle and cardiac motion. The aim of this study was to investigate the changes of RA function in patients with OSAS using VVI and to evaluate the application of VVI technology.

## Materials and methods

### Study population

We recruited 71 patients (age, 30–60 years; 30 females) diagnosed with OSAS for the first time after conducting overnight polysomnographies (PSG) at the sleep laboratory from October 2015 to March 2017. Thirty age and sex-matched healthy subjects (age, 30–60 years; 13 females) were enrolled after obtaining detailed history and performing physical examination, routine blood examination, electrocardiogram (ECG), overnight PSG, and transthoracic echocardiography (TTE). Written informed consent was obtained from all included subjects, which was approved by the institutional review board and ethics committee of the Qingdao University Medical School, Qingdao, China.

Sleep apnea refers to a reduction of ≥90% or complete obliteration in the respiratory airflow lasting ≥10s during sleep. Hypopnea was defined as a reduction ≥30% in the respiratory airflow lasting ≥10s and accompanied by a decrease ≥4% in oxygen saturation during sleep [13, 14]. The apnea–hypopnea index (AHI) was defined as the average number of episodes of apnea and hypopnea per hour of sleep. OSAS was defined as an AHI of > 5 per hour during sleep in the presence of clinical symptoms such as snoring, sleep fragmentation and upper airway obstruction. According to AHI, enrolled patients were classified into 3 groups: mild OSAS group (AHI = 5–15, *n* = 23), moderate OSAS group (AHI = 16–30, *n* = 25), and severe OSAS group (AHI > 30, *n* = 23).

The exclusion criteria were cardiac valve disease, coronary artery diseases, obstructive or restrictive lung disease, diabetes mellitus, connective-tissue diseases, atrial fibrillation, left ventricular (LV) systolic failure (ejection fraction < 50%), hypertension (systolic blood pressure > 140 mmHg and/or diastolic blood pressure > 90 mmHg). The subjects whose images were not clear could not be enrolled.

### Echocardiography

All patients underwent TTE using a commercially echocardiography system (Acuson SC2000 Ultrasound system; Siemens Medical Solutions, CA, with a 3.5 MHz probe). The images were obtained from the standard views in the left lateral position. The patients were advised to hold breath to obtain high-quality images to capture the entire RA. Averages of 3 consecutive cycles were measured for all echocardiograph data.

RA longitudinal diameter (RAL) and transverse diameter (RAT) were obtained in apical four chambers view. The RA volumes were obtained by tracing the RA endocardium at given time. The maximal RA volume (Vmax) was measured immediately before mitral valve opening. The minimal RA volume (Vmin) was measured precisely at mitral valve closure. The RA pre-a volume (Vpre) was measured at the precise beginning of the P wave in ECG. Right atrial volume parameters were corrected by body surface area (VImax, VImin, VIpre).The volumetric parameters of RA function were evaluated as follows:

RA reservoir function:

$$ \mathrm{Filling}\ \mathrm{volume}=\mathrm{Vmax}-\mathrm{Vmin}. $$

$$ \mathrm{RA}\ \mathrm{total}\ \mathrm{emptying}\ \mathrm{fraction}\ \left(\mathrm{RATEF}\right)=\left(\mathrm{Vmax}-\mathrm{Vmin}\right)/\mathrm{Vmax}\times 100\%. $$

RA conduit function:

$$ \mathrm{RA}\ \mathrm{passive}\ \mathrm{emptying}\ \mathrm{volume}\ \left(\mathrm{RAPEV}\right)=\mathrm{Vmax}-\mathrm{Vpre}-\mathrm{a} $$

$$ \mathrm{RA}\ \mathrm{passive}\ \mathrm{emptying}\ \mathrm{percent}\ \left(\mathrm{RAPEF}\right)=\left(\mathrm{Vmax}-\mathrm{Vpre}-\mathrm{a}\right)/\mathrm{Vmax}\times 100\%. $$

RA contraction function:

$$ \mathrm{RA}\ \mathrm{active}\ \mathrm{emptying}\ \mathrm{volume}\ \left(\mathrm{RAAEV}\right)=\mathrm{Vpre}-\mathrm{a}-\mathrm{Vmin} $$

$$ \mathrm{RA}\ \mathrm{active}\ \mathrm{emptying}\ \mathrm{percent}=\left(\mathrm{Vpre}-\mathrm{a}-\mathrm{Vmin}\right)/\mathrm{Vpre}-\mathrm{a}\times 100\%. $$

PAPs was determined by the following methods: PAPs = PTR + PRA. (PTR: tricuspid regurgitation pressure. PRA: RA pressure)

**Fig. 1:**
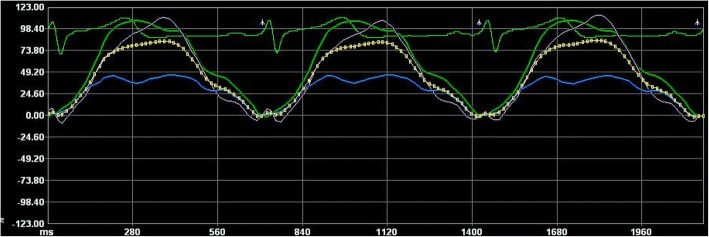
Right atrial strain determined by VVI. The strain of the atrial septum (green), RA roof wall (gray), lateral wall (blue) and RA-GLS (yellow) were measured

**Fig. 2:**
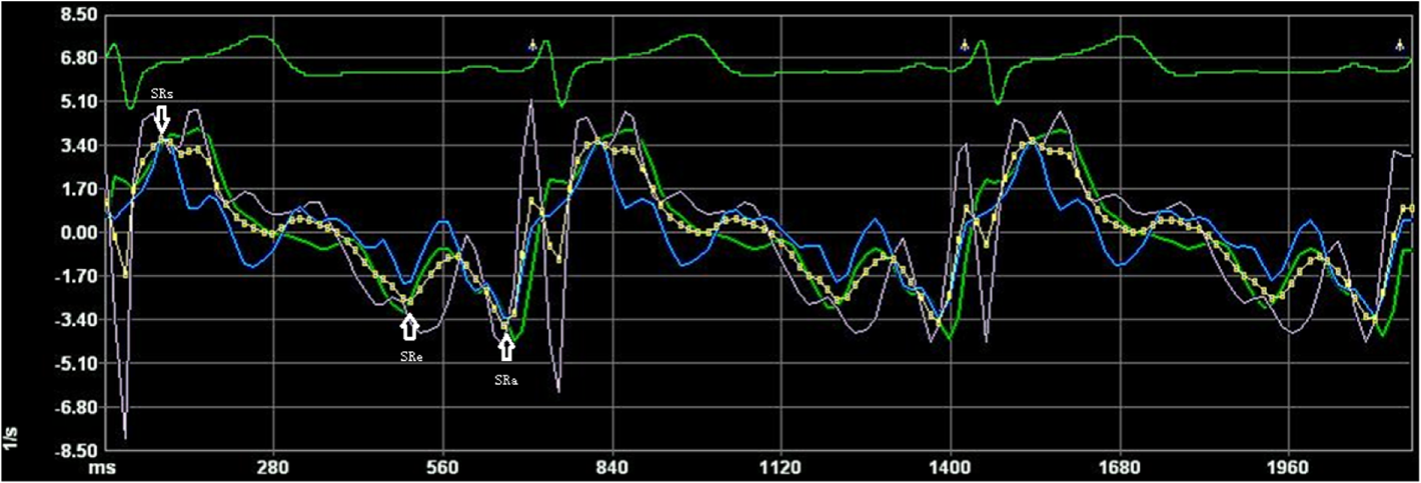
Right atrial strain rate determined by VVI. RA-SRs is the positive peak wave during ventricular systolic time (arrow). RA-SRe is the negative wave during early diastolic time (arrow). RA-SRa is the negative wave during late diastolic time (arrow)

For the assessment of RA function by VVI, Standard grayscale 2D images were obtained at a frame rate of 70–90 frames/s from apical 4-chamber images. Three cardiac cycles were stored at the end of expiration. At end systolic time, the RA endocardial border was traced manually from the medial portion of the tricuspid annulus, along with the atrial septum, RA roof wall, lateral wall and then back to lateral portion annulus. The RA epicardial border was determined by VVI software automatically. The region of interest was adjusted to include the entire myocardial wall. Longitudinal strain and strain rate curves were then generated for each atrial segment. The zero level was set at ventricular end-diastole. The RA peak atrial longitudinal strain (RA-PALS) was obtained from the strain curve. RA global longitudinal strain (RA-GLS) is the average of the RA-PALS of each segment (Fig. 1). The strain rate curve of the RA comprised one positive peak wave during ventricular systolic time, which means systolic longitudinal strain rate of RA (RA-SRs), one negative wave during early diastolic time, which means early diastolic longitudinal strain rate of RA (RA-SRe), and one negative wave during late diastolic time, which mean late diastolic longitudinal strain rate of RA (RA-SRa)(Fig. 2).

### Statistical analysis

All statistical data were analyzed using the SPSS version 18.0 software (SPSS Inc., IL) for Windows. The numeric variables were presented as mean ± standard deviation. Differences among continuous variables were performed using a one way analysis of variance (ANVOA). The chi-square test was used for comparison of data as appropriate. Pearson’s coefficient was used to determine the correlation between two variables. Multivariate regression analysis was used to eliminate the interaction among factors. A *P* value< 0.05 was considered significant.

## Results

### General information

The detailed demographic and clinical data of the 71 patients are presented in Table 1. The body mass index (BMI) was higher in the mild OSAS groups than in the healthy group (*t =* 4.19, *P* = 0.001); however, no differences were observed within the OSAS groups. The systolic pulmonary arterial pressure (PAPs) was higher in the severe OSAS group compared with control and mild groups (*t =* 2.76, *P* = 0.018; *t =* 3.478, *P* = 0.005, respectively). The variables of age, sex, systolic blood pressure (SBP), and diastolic blood pressure (DBP) showed no difference in the groups.

**Table 1:** Demographic and clinical characteristics of the groups

	Control(*n* = 30)	Mild(*n* = 23)	Moderate(*n* = 25)	Severe(*n* = 23)
Age(years)	46.82 ± 5.45	47.31 ± 6.15	47.96 ± 7.90	48.55 ± 5.43
Men/Women	17/13	13/10	14/11	14/9
BMI(Kg/cm^2^)	27.06 ± 4.38	28.40 ± 3.12	29.83 ± 5.05	32.97 ± 3.59^*^
SBP(mmHg)	122.19 ± 6.69	123.92 ± 7.08	126.27 ± 8.66	126.46 ± 10.65
DBP(mmHg)	73.51 ± 7.19	75.54 ± 6.43	76.11 ± 7.54	75.66 ± 8.47
AHI(events/h)	1.75 ± 0.99	13.96 ± 3.98^*^	24.01 ± 3.56^*†^	39.61 ± 6.64^*†‡^
HR(bpm)	71.55 ± 15.35	69.23 ± 11.57	70.01 ± 1.563	73.33 ± 13.04
PAPs(mmHg)	24.31 ± 5.63	25.44 ± 5.56	27.95 ± 5.61	33.80 ± 7.14^*†^

*AHI* Apnea–hypopnea index, *BMI* body mass index, *DBP* diastolic blood pressure, *SBP* systolic blood pressure, *HR* heart rate, *PAPs* systolic pulmonary arterial pressure

^*^P < 0.05 vs controls; †*P* < 0.05 vs mild; ‡*P* < 0.05 vs moderate

### Conventional echocardiographic parameters

The conventional echocardiograph data are demonstrated in Table 2. The data of RAL and RAT in severe OSAS group were higher compared with control group (*t =* 2.498, *P* = 0.022; *t =* 2.245, *P* = 0.033, respectively). The values of VImax were higher in moderate and severe OSAS groups than that of control group (*t =* 2.237, *P* = 0.047; *t =* 2.469, *P* = 0.031, respectively). The values of VImin, VIpre in severe OSAS group were increased compared with control group (*t =* 2.326, *P* = 0.044; *t =* 2.405,*P* = 0.035, respectively). RATEF showed no difference in the groups. RAPEF showed lower in moderate and severe OSAS groups than in control group(*t =* 2.681,*P* = 0.021;*t =* 2.985,*P* = 0.011, respectively). RAAEF showed increased in moderate OSAS group compared with control group (*t =* 3.006, *P* = 0.02), but showed no difference in severe OSAS and control groups (*P* > 0.05).

**Table 2:** Conventional echocardiographic parameters in different groups

	Control(*n* = 30)	Mild(*n* = 23)	Moderate(*n* = 25)	Severe(*n* = 23)
RAL(mm)	4.13 ± 0.67	4.31 ± 0.67	4.44 ± 0.66	4.81 ± 0.78^*^
RAT(mm)	3.67 ± 0.73	3.90 ± 0.84	4.22 ± 0.72	4.54 ± 0.83^*^
VImax(ml/m^2^)	24.9 ± 4.17	26.9 ± 6.69	29.0 ± 5.75^*^	30.1 ± 6.15^*^
VImin(ml/m^2^)	11.0 ± 2.59	10.9 ± 3.56	12.7 ± 4.01	14.6 ± 4.54^*^
VIpre(ml/m^2^)	16.7 ± 3.34	17.5 ± 3.25	18.1 ± 2.88	19.8 ± 3.23^*^
RATEF (%)	67.8 ± 7.74	68.8 ± 6.43	69.3 ± 6.75	70.5 ± 7.98
RAPEF (%)	45.1 ± 7.00	41.4 ± 7.22	37.5 ± 6.33^*^	36.0 ± 7.87^*^
RAAEF (%)	53.6 ± 6.67	54.7 ± 6.72	58.8 ± 8.04^*^	57.9 ± 5.94

^*^P < 0.05 vs controls; †*P* < 0.05 vs mild; ‡*P* < 0.05 vs moderate

*RAL* RA longitudinal diameter, *RAT* RA transverse diameter, *VImax* RA maximum volume index, *VImin* right atrial minimum volume index, *VIpre* right atrial volume index before contraction, *RATEF* right atrial total emptying fraction, *RAPEF* right atrial passive emptying fraction, *RAAEF* right atrial active emptying fraction

### RA function using VVI

The VVI data of RA are presented in Table 3. The values of RA-GLS were reduced in moderate OSAS group compared with control group (*t =* 2.333, *P* = 0.040), and reduced more obviously in severe OSAS group (vs control: *t =* 3.25, *P* = 0.008; vs mild: *t =* 3.011, *P* = 0.012, respectively). RA-SRs in severe OSAS group showed decreased compared with control and mild groups (*t =* 3.665, *P* = 0.004;*t =* 3.204,*P* = 0.008, respectively). RA-SRe in moderate and severe groups were decreased compared with control group (*t =* 2.466, *P* = 0.031; *t =* 3.547, *P* = 0.005, respectively). RA-SRa in severe OSAS group OSAS were decreased compared with control group (*t =* 2.425, *P* = 0.034).

**Table 3:** VVI data of RA in different groups

	Control(*n* = 30)	Mild(*n* = 23)	Moderate(*n* = 25)	Severe(*n* = 23)
RA-GLS(%)	41.6 ± 7.7	36.5 ± 6.5	33.6 ± 8.2^*^	30.5 ± 7.8^*^†
RA-SRs(1/s)	2.21 ± 0.63	2.17 ± 0.61	1.88 ± 0.63	1.51 ± 0.58^*^†
RA-SRe(1/s)	− 1.97 ± 0.62	− 1.54 ± 0.63	−1.30 ± 0.53^*^	− 1.18 ± 0.64^*^
RA-SRa(1/s)	− 2.18 ± 0.55	− 2.21 ± 0.68	− 1.80 ± 0.58	−1.56 ± 0.60^*^

*RA-GLS* RA global longitudinal strain, *RA-SRs* systolic longitudinal strain rate of RA, *RA-SRe* early diastolic longitudinal strain rate of RA, *RA-SRa* late diastolic longitudinal strain rate of RA

^*^P < 0.05 vs controls; †*P* < 0.05 vs mild; ‡*P* < 0.05 vs moderate

### Correlation analysis

**Fig. 3:**
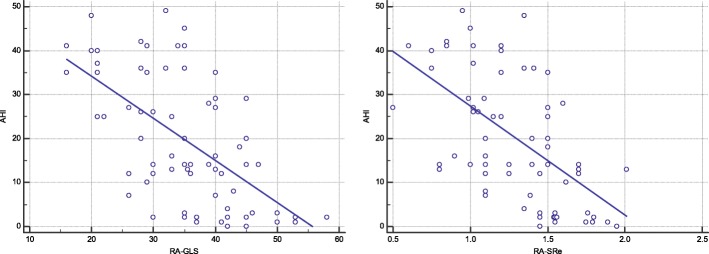
Correlation between AHI and RA-GLS (*r* = − 0.593,*P*<0.001), RA-SRe (*r* = − 0.565,*P* = <0.001)

The results of the correlation analyses of RA functional indices with AHI were presented in Table 4 and Fig. 3. To eliminate the interaction among factors, multivariate regression analyses was used, and the results showed RA-GLS and RA-SRe were independently correlated with AHI (Table 5).

**Table 4:** The correlation analyses of RA functional indices with AHI

	*r*	*P*
RA-GLS	−0.593	< 0.001
RA-SRs	−0.379	0.007
RA-SRe	−0.565	< 0.001
RA-SRa	−0.381	0.007
RAL	0.382	0.007
RAT	0.37	0.01
VImax	0.38	0.022
VImin	0.357	0.013
VIpre	0.273	0.06
RATEF	0.148	0.315
RAPEF	−0.386	0.007
RAAEF	0.14	0.451

**Table 5:** The multivariate regression analyses of RA functional indices with AHI

	Coef.	Std. Error	Beta	*t*	*P*
RA-GLS	−0.509	0.186	−0.425	−2.738	0.010
RA-SRe	−4.569	2.086	−0.295	−2.191	0.039

## Discussion

We evaluated RA function by VVI compared with conventional echocardiograph parameters. No obvious difference was found in mild OSAS and control group. In moderate OSAS group, RA VImax and RAAEF were higher, but RAPEF was lower than control group (*t =* 2.237, *P* = 0.047; *t =* 3.006, *P* = 0.02; *t =* 2.681, *P* = 0.021, respectively), which suggested that RA reservoir and contraction function was increased, and conduit function was decreased. In patients with OSAS, respiration efforts against the increased upper airway resistance increase the intrathoracic negative pressure and venous return, leading to increased RV preload [15]. Consequently, RA reservoir function was increased. Meanwhile, RV dysfunction resulting from ischemia and structural remodeling [16] leads to the increase of the RV end diastolic pressure. The pressure gradient between RA and RV decreased at the end of diastole. RAPEF began to be lower than that of the control group (*t =* 2.681, *P* = 0.021), suggesting that the RA conduit function was reduced. More blood would be stored in RA before contraction. It has been proved that Frank-Starling mechanism is also applicable to RA [11].That is, when the increase in RA preload elongates the myocardium, the myocardial contractility is enhanced, so the RA contraction function is increased. RAAEF in OSAS moderate group is higher than that of control group (*t =* 3.006, *P* = 0.02). Therefore, the blood filling of RV is sufficient in patients with OSAS although RV diastolic dysfunction. Willens HJ [17] et al. also verified the compensatory mechanism in the study of patients with different stages of pulmonary hypertension. In severe OSAS group, with the development of the disease, RV diastolic and systolic function is further reduced, and the RA VImax is further increased (vs. control group: *t =* 2.469, *P* = 0.031). RA VImin and VIpre also increased (vs. control group: *t =* 2.326, *P* = 0.044; *t =* 2.405, *P* = 0.035, respectively). RAPEF was further reduced (vs. control group: *t =* 2.985, *P* = 0.011), suggesting a further reduction of RA conduit function. But RAAEF was no difference in OSAS severe group compared with the control group, which may indicate that the compensatory ability of RA myocardium has reached the maximum or near the edge of decompensation.

Compared with conventional echocardiography, VVI has obvious advantages in evaluating the function of RA which is with irregular shape because VVI technology is independent of the geometric shape of the heart cavity [18]. In this study, RA-GLS began to decrease in moderate OSAS group (vs. control group: *t =* 2.333, *P* = 0.040), and decreased more obviously in severe OSAS group (vs. control group: *t =* 3.25,*P* = 0.008;vs. mild OSAS group: *t =* 3.011,*P* = 0.012), which suggests the reduction of RA myocardial deformability. Some investigators consider that RA-GLS represents the RA reservoir function. But we consider that the increase of RA reservoir function is reflected in the fact that RA can receive more venous blood flow in the systolic period. However, RA-GLS represents the deformation ability of the RA myocardium, which is not equal to RA reservoir function. In the patients with OSAS, RA enlarged and RA reservoir function increased. It might be due to the excessive extension of RA myocardium, which reduces its deformation capacity and damages its function. The RA-SRe in the moderate OSAS group was lower than that of the control group (*t =* 2.466, *P* = 0.031), suggesting that RA function began to decrease at the early diastolic period, which was consistent with the decrease of RA conduit function. RA conduit function, that is, the ability of passive emptying, mainly depends on the active relaxation of RV. The right ventricular active relaxation ability decreased in patients with OSAS because of the remodeling, thickening and relative ischemia of right ventricular wall [19], which resulted in slow filling of the blood flow back to RV, that is, RA conduit function was decreased.

In the severe OSAS group, RA-GLS and RA-SRe were further reduced, and RA-SRs and RA-SRa began to decrease (RA-SRs: vs. control group *t =* 3.665, *P* = 0.004;vs. mild OSAS group *t =* 3.204, *P* = 0.008; RA-SRa: vs. control group *t =* 2.425,*P* = 0.034), suggesting further impairment of RA function. This is associated with further impairment of RA diastolic and systolic function. In addition, RA overexpansion, myocardial fibrosis and remodeling of atrial wall can also decrease the function of RA [20]. Multivariate regression analysis showed RA-GLS and RA-SRe directly affect the AHI (*t =* − 2.738, *P* = 0.010; *t =* − 2.191, *P* = 0.036; respectively), suggesting that RA-GLS and RA-SRe were independent predictors of disease severity in patients with OSAS.

### Limitations

A technical limitation of our study was that the VVI method was depended on the grayscale (B-mode) images, which was affected by the pulmonary conditions or the presence of obesity in patients with OSAS. The image quality could be improved by breath holding and selecting acoustic mood to acquire images. Another salient drawback in the present study is the small sample size. A larger longitudinal study would be necessary to confirm the results obtained in this study.

## Conclusion

RA function was reduced to varying degrees in OSAS patients, which occurred before the beginning of pulmonary hypertension and heart failure. On hemodynamics, the change of right atrial function performed increased of reservoir function, reduced conduit function and increased of contraction function. However, the strain and strain rate reduced in different degree. RA-GLS and RA-SRe decreased the earliest, which suggested that strain and strain rate were the parameters which can reflect myocardial function damage earliest. OSAS patients with reduced RA-GLS and RA-SRe can be given intervention as early as possible. RA-GLS and RA-SRe also can be used as one of the indicators of the effectiveness of treatment. RA-GLS and RA-SRe were directly correlated with AHI, suggesting that RA-GLS and RA-SRe were independent predictors of disease severity in patients with OSAS. VVI can earlier and accurately detect myocardial dysfunction of RA in patients with OSAS, which is expected to be a worthy technique for early clinical therapy in patients with OSAS.

## Abbreviations

AHI: Apnea-hypopnea index
BMI: Body mass index
OSAS: Obstructive sleep apnea syndrome
PAPs: Systolic pulmonary arterial pressure
RA: Right atrium
RAAEF: Right atrial active contraction emptying fraction
RA-GLS: Right atrial global longitudinal strain
RAL: RA longitudinal diameter
RAPEF: Right atrial passive emptying fraction
RA-SRa: Right atrial strain rate in ventricular late diastolic phase
RA-SRe: Right atrial strain rate in ventricular early diastolic phase
RA-SRs: Right atrial strain rate in ventricular systolic phase
RAT: RA transverse diameter
RATEF: Total right atrial emptying fraction
Vmax: RA maximum volume
Vmin: RA minimum volume
Vpre: Right atrial volume before contraction
VVI: Velocity vector imaging
